# A Parallel Mediation Model of Career Adaptability, Career Self-Efficacy, and Future Career Choice Among University Students: The Role of Basic Psychological Needs Satisfaction and Mindfulness

**DOI:** 10.3390/ejihpe15040047

**Published:** 2025-03-26

**Authors:** Girum Tareke Zewude, Anmut Endalkachew Bezie, Getachew Tassew Woreta, Tsehaynew Getaneh Tareke, Tun Zaw Oo, Ayalew Hassen, Merkebu Tareke, Yvette Orsovics, Krisztián Józsa

**Affiliations:** 1 Department of Psychology, Wollo University, Dessie 1145, Ethiopia; getachew.tassew@wu.edu.et; 2 Department of Occupational Health and Safety, College of Medicine and Health Sciences, Wollo University, Dessie 1145, Ethiopia; anmut.endalkachew@wu.edu.et; 3 Doctoral School of Education, University of Szeged, 6722 Szeged, Hungary; tsehaynew.getaneh.tareke@edu.u-szeged.hu; 4 Institute of Education, Hungarian University of Agriculture and Life Sciences, 7400 Kaposvár, Hungary; oo.tun.zaw@uni-mate.hu; 5 MTA-MATE Early Childhood Research Group, Hungarian University of Agriculture and Life Sciences, 7400 Kaposvár, Hungary; 6 Department of Curriculum and Instruction, Wollo University, Dessie 1145, Ethiopia; ayalewhassen17@gmail.com; 7 Department of Urban and Infrastructure Development, South Wollo Zone, Dessie 1145, Ethiopia; merkebutareke2005@gmail.com; 8 Department of Primary and Pre-School Education, J. Selye University, 94501 Komarno, Slovakia; orsovicsy@ujs.sk; 9 Institute of Education, University of Szeged, 6722 Szeged, Hungary

**Keywords:** career adaptability, self-efficacy, basic psychological needs satisfaction, mindfulness, career choice, parallel mediation analysis

## Abstract

This study aims to explore the importance of basic psychological needs satisfaction (BPNS) and mindfulness of university students in their career adaptability (CA), career self-efficacy (CSE), and future career choice (CC). The sample consisted of 1026 undergraduate students, of which 495 (48.2%) were female and 531 (51.8%) male, from three public universities in the Amhara Region, Ethiopia. The assessments, such as the Career Decision-Making Self-Efficacy Scale-Short Form, the Five Facet Mindfulness Questionnaire Short Form, the Career Adapt-Ability Short Form Five Scale, the Basic Psychological Needs Satisfaction Scale, and the Career Choice scale instruments were used. Findings from a parallel mediation model showed that both CA and CSE had a direct positive effect on the BPNS of university students. Additionally, this study found that both BPNS and mindfulness had a substantial and positive direct effect on the students’ CC. Importantly, we found that both CA and CSE significantly and indirectly predicted the future CC through BPNS and mindfulness. In addition, BPNS and mindfulness also partially mediate the relationship between CA, CSE, and the students’ future CC. Importantly, we found that mindfulness and BPNS fully and partially mediated the relationship between CA, CSE, and CC. These results support the notion that both psychological constructs (BPNS and mindfulness) play a crucial role in explaining the relationship between CA, CSE, and CC. By enhancing students’ BPNS and mindfulness, institutions can empower students to make informed decisions about their future career paths, ultimately nurturing their personal and professional growth.

## 1. Introduction

Nowadays, the job market and economic conditions, such as a decreasing unemployment rate and limited job demands, pose significant challenges for the graduates of universities and colleges in Ethiopia (Tareke et al., 2024). Unmet psychological needs can significantly affect graduates’ overall well-being and career outcomes. Research indicates that when basic psychological needs are not met, it can lead to decreased motivation and engagement in career-related activities (Xu et al., 2025). Furthermore, factors contributing to the lower level of motivation among students—such as a lack of support, unclear career pathways, and external pressures—can hinder academic performance and career decision-making (Raboca & Carbunarean, 2024). Additionally, students often face challenges in making informed career choices due to limited access to career guidance and information about job markets, which can result in poor decision-making regarding their fields of study (Xu et al., 2025). Lastly, the absence of essential career adaptability skills can hinder graduates’ ability to navigate the job market effectively, as studies have shown that those lacking these skills often struggle to adjust to changing job demands and career paths (Bocciardi et al., 2017).

A lack of mindfulness or positive understanding towards their future career life also contributes to difficulties in finding employment and improving their economic status (Zewude et al., 2024a). These challenges can also impact an individual’s future career, and they are faced with numerous challenges and uncertainties when it comes to making CC (Santana-Monagas & Núñez, 2022). In Ethiopia, CC is the biggest problem among undergraduate students. A previous study indicates that a significant majority of students (70.7%) entered their fields not by choice, but rather due to various external pressures, such as familial expectations, socioeconomic constraints, and limited access to information about diverse career options (Getachew, 2022; Kumsa et al., 2020). These influences often lead students to pursue majors that do not align with their personal interests or long-term career aspirations. For instance, familial expectations may pressure students into fields perceived as more prestigious or financially stable, while socioeconomic factors can restrict their choices to more readily accessible programs (Abrahams et al., 2015; Getachew, 2022). Furthermore, the lack of comprehensive information regarding potential career paths can result in uninformed decision-making, ultimately impacting students’ career trajectories. Understanding these dynamics is crucial for addressing the challenges students face in aligning their educational pursuits with their career goals.

For students, choosing a career is a difficult decision because it affects the type of career they want to follow in the future (Njeri, 2013). Universities and educational institutions can help students overcome these challenges by providing support, resources, and a supportive learning environment that meets their psychological needs and fosters soft skill development. Three important constructs that have gained attention in the field of career development are CA, career self-efficacy (CSE), and future CC (Mini et al., 2023). CA refers to an individual’s capacity to effectively manage career-related transitions (Savickas & Porfeli, 2012; Sou et al., 2021), while CSE pertains to an individual’s belief in their ability to perform successfully in a given career domain (Lomas et al., 2017). Hence, understanding the factors that influence these constructs is essential for promoting informed future CCs and facilitating successful career outcomes.

In addition to academic and technical skills, students need to develop soft skills such as CA, CSE, and basic psychological needs strategies. Mindfulness skills improve CCs (Stead et al., 2022). This can help them find employment, adapt to changing job market conditions, and pursue fulfilling careers. Scientific literature evidence that CA, future CSE, basic psychological needs of satisfaction, and mindfulness will enhance CC and can play crucial roles in reducing poverty through education (Chevrier & Lannegrand, 2021). By producing qualified university and college graduates who possess the necessary skills and knowledge to succeed in the job market, education can help individuals improve their economic status and reduce poverty (Chevrier & Lannegrand, 2021; Gülşen & Şahin, 2022). Furthermore, fostering the psychological needs of learners, such as a sense of competence, autonomy, and relatedness, can increase their motivation to learn, foster CA and CSE, enhance their CC, and enhance their overall well-being (Deci & Ryan, 2000). This, in turn, can help students stay engaged in their studies, persist in their educational pursuits, develop CA skills, and lead to greater success, hope, and meaning in life in the long run. 

Despite the extensive research that has been conducted on CA (Hirschi et al., 2015; Merino-Tejedor et al., 2016; Savickas & Porfeli, 2012), basic psychological needs (Burgueño et al., 2023; Deci & Ryan, 2000; Y. Wang et al., 2019), CSE and CC (Chan, 2018), and mindfulness (Hou et al., 2014; Yagil et al., 2023), there are still several significant research gaps that need to be addressed. Firstly, the current educational landscape in Ethiopia may hinder the development of CA skills among young people, potentially impacting their confidence, control, curiosity, concern, and cooperation as future graduates. Without a clear vision of their future, students may struggle to set goals and develop clear planning as well as problem-solving skills or CSE, which can lead to a lack of motivation and productivity in their professional and personal lives. This can result in a lack of CA, a lack of mindfulness skills, and a lack of autonomy, competence, and CC, which can further impact their future (Gülşen & Şahin, 2022). Secondly, a lack of CA and CSE can also contribute to a cycle of poverty and inequality. When students are unable to find suitable jobs, they may struggle to achieve financial stability and meet their basic needs, which can lead to a lack of opportunities for career advancement and personal growth. Thirdly, while there are some previous research studies examining the relationship between the constructs of basic psychological need, CSE, CA, mindfulness, and CC (e.g., Gülşen & Şahin, 2022; Hirschi et al., 2015; Merino-Tejedor et al., 2016; Y. Wang et al., 2019; Zhang et al., 2022), the links have not been adequately researched, and therefore, it is timely to examine this relationship concerning these essential career-related elements that are essential for students to thrive and feel fulfilled in their future careers.

This study explicitly addresses these gaps by deepening our understanding of the complex relationship between these psychological and vocational-related constructs and sheds light on the role that basic psychological needs and mindfulness play in mediating this relationship. This is also a unique lens to examine how career adaptability, self-efficacy, and mindfulness function in resource-constrained, collectivist societies (e.g., Ethiopia). By exploring these factors, this study contributes to global career development literature. Moreover, it provides actionable strategies for universities to set interventions that align with students’ psychological and cultural needs.

## 2. Literature Review

### 2.1. Career Adaptability, Career Self-Efficacy, and Mindfulness

Studies indicate a positive relationship between career adaptability (CA), mindfulness, and career self-efficacy (CSE). CA, defined as the ability to navigate and adjust to career-related changes, is a crucial factor in career decision-making and success (Savickas, 2005). Another influential factor in CC is mindfulness, defined as a non-judgmental awareness of the present moment (Baer et al., 2006). Recent studies that support this finding include the research conducted by Guan et al. (2013) with a sample of Chinese university students to explore the relationship between CA and CSE. In addition, a study by Savickas (2005) found that individuals with higher levels of CA exhibited greater career decision-making self-efficacy. Similarly, another study demonstrated that CA positively influenced CSE beliefs among young adults (Hirschi & Valero, 2015). Mindfulness can help to buffer the negative effects related to low CA by building resilience to work stress. 

Recent studies have underscored the importance of mindfulness in enhancing adaptability and academic achievement. A 2024 study found that mindfulness positively impacts academic performance through its mediating role in adaptability among students, indicating that mindfulness fosters both personal well-being and adaptability in academic and career contexts (Bordbar et al., 2024). Additionally, mindfulness helps mitigate the negative effects of social media pressures, enabling individuals to manage stress and adapt to changing social dynamics, which is crucial for success in modern careers (You & Liu, 2022). Furthermore, adaptability has been shown to mediate the relationship between mindfulness and various outcomes, including career success, emphasizing the need to cultivate mindfulness to enhance adaptability in professional settings (Bordbar et al., 2024).

Career adaptability is a person’s psychosocial resources, which are required to deal with the unstable career landscape of contemporary society, especially for university and college students through managing unfamiliar tasks and transitions (Amalia & Kurniawati, 2019) and it is better to treat it using mindfulness strategies (Baer et al., 2006). These findings suggest that more adaptable individuals tend to have higher levels of confidence in their ability to make career-related choices. In addition, students with high and medium levels of CA are more comfortable and clearer about their future CC (Mahmud & Adira Khidir, 2023). It is an essential tool for university students since it adds essential value for students to successfully transition to employment and improve their quality of life by relating with positive variables such as higher employment quality, career satisfaction, general and professional well-being, as well as self-regulation, career construction, and academic engagement (Lee & Jung, 2022).

CSE refers to an individual’s belief in their ability to succeed in various career-related tasks, which significantly influences their motivation and decision-making (Haenggli & Hirschi, 2020). It is also an influential predictor of CC and mindfulness, which refers to an individual’s belief in their capabilities and competence to successfully perform and excel in various future career-related tasks and activities (Lent et al., 1994). The concept is derived from Albert Bandura’s social cognitive theory, which emphasizes the role of self-beliefs in human motivation, behavior, and achievement (Bandura, 1982). High levels of self-efficacy empower individuals to adapt to changing circumstances, enhancing their CA—the capacity to adjust one’s career plans and strategies in response to challenges and opportunities (Haenggli & Hirschi, 2020). This adaptability is crucial for making an informed and confident CC, ultimately leading to greater satisfaction and fulfillment in one’s professional life (Gushue et al., 2006). A feeling of self-efficacy allows individuals to choose their career path and adapt in the transition period and has positive effects on job performance (Savickas & Maggiori, 2017).

Recent studies have highlighted the potential role of CSE in boosting mindfulness. A study by Lomas et al. (2017) found that individuals who have a high level of mindfulness may also have greater self-efficacy beliefs, leading to more informed and confident CCs. The study shows that there is no significant difference between CSE and gender (Mahmud & Adira Khidir, 2023). Previous literature shows that excellent results can be attained by students with high self-efficacy compared to those with low self-efficacy (Mahmud & Adira Khidir, 2023). The study’s findings demonstrated that poor CSE causes cognitive disorders connected to one’s career and impairs one’s ability to make wise judgments (Bullock-Yowell et al., 2012). Conversely, those who have a high level of CSE are more assured of their ability to make choices and choose a course for their career. CSE is the cornerstone of CC (Komarraju et al., 2014). Students with low levels of self-confidence and awareness lack confidence in themselves when choosing a career, lack understanding about the career they want to pursue, and choose a less suitable career. 

### 2.2. Career Adaptability, Career Self-Efficacy and Basic Psychological Needs Satisfaction

Career adaptability (CA) is a vital construct in the field of career development, referring to an individual’s ability to effectively navigate career-related challenges and transitions (Savickas, 2005). It also describes a person’s ability to cope with current and future tasks, changes, and career traumas. This construct comprises vital resources like concern, control, interest, and confidence, which indicate self-regulation abilities or capacities for dealing with job obstacles (Matijaš & Seršić, 2021). Understanding the factors that contribute to CA is important for boosting successful career decision-making and adjustment. Self-determination theory (SDT) is the one global theoretical framework that provides a useful role in examining the role of basic psychological needs satisfaction (BPNS) in CA (Deci & Ryan, 2000). According to SDT, individuals have three fundamental psychological needs: competence, autonomy, and relatedness. Satisfying these needs is essential for optimal motivation, well-being, and performance. Recent research found that CA and basic psychological needs have positive relationships (Şahin & Gülşen, 2022). This means that the higher CA the students have, the more satisfied with their basic psychological needs.

Career self-efficacy (CSE) plays a significant role in influencing basic psychological needs satisfaction (BPNS) among university students. For example, in a study (Basileo et al., 2024) investigating the role of students’ CSE and BPNS in academic achievement, it was found that students’ CSE had a statistically significant association with their BPNS and had a reciprocal relationship with both autonomous and controlled motivation. Moreover, van Zyl and Dhurup (2018) studied self-efficacy and its relationships with satisfaction with life and happiness among university students. Their study found in both genders that if students have higher self-efficacy scores, they have higher satisfaction in life and happiness. In their study, self-efficacy generally provides serious satisfaction to the students’ lives. Additionally, Macakova and Wood (2022) found that university students’ self-efficacy is positively associated with the satisfaction of basic psychological needs. Therefore, it can be seen that self-efficacy and BPNS have positive relationships among university students.

### 2.3. Mindfulness, Career Self-Efficacy, and Career Choice

Mindfulness may enhance students’ career choice (CC) by making them aware of their interests and abilities. This provides them with an increase in readiness through an increase in occupational information, generalized self-efficacy, and fewer perceived barriers, which helps students in their CC. The existing literature suggested that both mindfulness and self-efficacy have a positive effect on performance and CC due to the enhanced effect mindfulness and self-efficacy have on performance, such as perseverance and reducing stress that inhibits performance (Yagil et al., 2023). Mindfulness may help in CC by reducing stress and enhancing emotional awareness, control, and coping with experiences with openness and acceptance (Charoensukmongkol & Puyod, 2022). Mindfulness has a positive relationship with self-efficacy; increasing mindfulness leads to a rise in self-efficacy. Studies show that mindfulness is positively related to self-efficacy, job performance, and satisfaction and ends in positive outcomes such as improved task performance, decreased turnover intentions, and emotional exhaustion by being positively associated with high levels of alertness and cognitive flexibility (Aydoğmuş, 2022). Being mindful helps people improve their self-efficacy because they are more able to regulate and control their emotions and assess how they see themselves about the people, places, things, and ideas in their work environment (Aydoğmuş, 2022).

A workplace study demonstrated that mindfulness can improve the performance and well-being of individuals by managing stress, burnout, pain, depression, anxiety, anger, and addiction, reducing emotional reactivity to negative events, and reducing deviance from safety issues (Johnstone & Wilson-Prangley, 2021). Moreover, mindfulness can improve various aspects of adaptability, related to career, a crisis, uncertainty, learning, and problem-solving (Johnstone & Wilson-Prangley, 2021). CSE has a direct relationship with students’ CC. Self-efficacy influences a person’s choice of career by reducing the negative effects of stress because those with high levels of self-efficacy tend to welcome challenges and see obstacles as opportunities for growth (M. Li et al., 2023). Students with strong self-efficacy can confidently overcome obstacles in their professional progress and make an effort to reach their goals (Chan, 2018). Self-efficacy influences college students’ perceptions of their ability to make wise career decisions (Nachmias & Walmsley, 2015). College students who have higher levels of self-efficacy are more likely to make wise CC and explore their options (Chan, 2018; Nachmias & Walmsley, 2015).

### 2.4. Career Adaptability, Career Choice, Mindfulness, and Basic Psychological Needs Satisfaction

Career adaptability (CA) affects CC both directly and indirectly through self-efficacy in making career decisions (Stead et al., 2022). According to a study in China, career knowledge and adaptability have influenced students’ CC by facilitating individuals’ efficacy in making CC decisions (Stead et al., 2022). People who lack confidence when making CCs are more likely to struggle and be dissatisfied with the results of their decisions (Kulcsár et al., 2020). The basic psychological needs of satisfaction plays an important role as a guiding factor that affects students’ CA (Xu et al., 2023). When the students’ basic psychological needs and satisfaction increase, their level of CA also increases. Students with high CA are encouraged to focus on their career development, stimulate curiosity about potential careers, and foster career control and self-confidence. Conversely, if their basic psychological needs are low or unmet, it can have negative effects on their career development. Basic psychological needs, such as self-relatedness, autonomy, and alertness, are positively related to career self-efficacy and career choice. Basic psychological needs might boost their self-confidence in their capacity to organize their career and resolve employment-related issues (Chan, 2018). According to the self-determination theory (SDT), students who have a basic psychological need can regulate themselves, experience psychological freedom and self-choices, interact effectively with the learning environment, master challenging activities, have a good feeling of relationship with lectures, follow their friends, experience connection with their lecturers and fellow students, and experience friendliness (Benlahcene et al., 2020). This leads students to pay more attention, feel interested, use in-depth learning strategies, express their thoughts, and face their career challenges, such as undertaking coursework, progressing in their studies, striving for a degree and a career, fulfilling unexpected requirements, and achieving their choice of career (Benlahcene et al., 2020). As a mediator, students’ mindfulness plays a significant role in the relationship between career adaptability and career choice, as well as between psychological flexibility and well-being. In a study (de Abreu et al., 2024), mindfulness did not have a direct relationship with CA. Nevertheless, as students are mindful, they have a strong vocational identity, which can predict their career adaptability. A study (Galles et al., 2019) involving undergraduate students found that mindfulness was significantly associated with fewer negative career thoughts, specific decision-making styles, and higher vocational identity. This suggests that mindfulness may play a role in shaping the thoughtful processes and self-identity related to career choices. 

### 2.5. Theoretical Foundations and Research Hypothesis

The relationship between CA, CSE, mindfulness, BPNS, and university students’ future career choice is intricately associated with the theoretical frameworks of positive psychology (Seligman & Csikszentmihalyi, 2000), the social cognitive career theory (Hackett & Betz, 1980), career construction theory (Savickas, 2005), and self-determination theory (Deci & Ryan, 2000). 

The positive psychology theory (PPT) is one of the best positive resource-oriented theories, which explains how CA and CSE can enhance individuals’ well-being and satisfaction in CC (Ladnai, 2019; Seligman & Csikszentmihalyi, 2000). CSE, CA, and CC are interrelated concepts that align closely with the principles of positive psychology. By fostering a positive psychological framework, individuals can strengthen their self-efficacy and adaptability, enabling them to navigate their career paths more effectively and make choices that align with their strengths and aspirations, thereby enhancing overall well-being and success (Gushue et al., 2006). By emphasizing mindfulness and resilience, positive psychology underscores the significance of these constructs in shaping students’ future career trajectories (Galles et al., 2019; Yagil et al., 2023). Secondly, this study’s argument derives from the career construction theory (CCT) (Savickas, 2005). CCT offers insights into how individuals construct their career paths through storytelling and identity formation (Savickas, 2005). CA and CSE play crucial roles in shaping these narratives, guiding students’ perceptions of career options, decision-making processes, and transitions throughout their academic and professional journey (Rudolph et al., 2017). Thirdly, another comprehensive theoretical foundation used in this study was the social cognitive career theory (SCCT) (Hackett & Betz, 1980). This theory is pivotal as it explores the interconnectedness of CA, CSE, and students’ future CC. CSE, rooted in Bandura’s theory, influences individuals’ belief in their ability to navigate tasks, while CA enables them to adapt to changing circumstances (Stead et al., 2022). Mindfulness practices, promoting self-awareness and emotional regulation, may bolster CSE and adaptability. Together, these elements form a complex web influencing university students’ CC, underlining the significance of a holistic approach in fostering successful career development and decision-making processes (Haenggli & Hirschi, 2020). The authors also focused on the increasing dynamics of careers, making the development and application of various career resources important for successful career development (Haenggli & Hirschi, 2020). Thus, focused on the practical importance of SCCT, Lent and Brown (2020) believe that people have varying degrees of agency in their career decision-making processes, and along with outcome expectations and personal goals, CSE plays an important role (Stead et al., 2022). Therefore, CSE and CA are the potential independent factors that determine students’ future CC. Fourthly, self-determination theory (SDT) provides a framework to understand intrinsic motivation and psychological needs (autonomy, competence, and relatedness) driving human behavior (Deci & Ryan, 2000). CA and CSE are viewed as factors enhancing autonomy, competence, and relatedness, empowering students to pursue their career goals with confidence whilst supporting the fulfillment of their basic psychological needs (Şahin & Gülşen, 2022).

**Figure 1:**
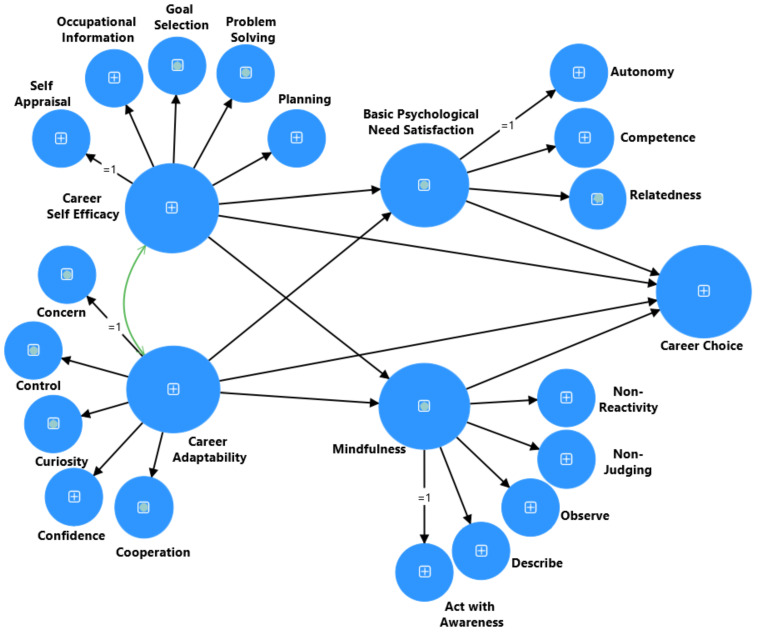
A Proposed full parallel mediation model to explain the association between CA, CSE, BPNS, mindfulness, and CC.

By integrating insights from positive psychology, the social cognitive career theory (SCCT), career construction theory, and self-determination theory, researchers and practitioners can develop a holistic understanding of the motivational factors and psychological processes influencing career decision-making among students. These theories emphasize the importance of BPNS and mindfulness as driving needs and positive psychological resources for boosting CA and CSE, fostering an informed and satisfying CC. To our knowledge, this study is pioneering in exploring the role of parallel mediation involving CA, CSE, and university students’ future CC. It examines the impacts of BPNS and mindfulness from the perspective of a developing country. Grounded in strong evidence from recent scientific literature and the conceptual framework which is demonstrated in Figure 1, this study seeks to address the following research questions:

RQ1: What is the relationship between socio-demographic factors and students’ CA, CSE, BPNS, mindfulness, and future CC?

RQ2: What is the relationship between students’ CA, CSE, BPNS, mindfulness, and future CC?

RQ3: Do the students’ CA and CSE directly or indirectly (via BPNS and mindfulness) influence students’ future CC? 

## 3. Materials and Methods

### 3.1. Sample and Sampling

The samples (1026 students) were selected from public universities in the Amhara Regional State of Ethiopia (Table 1). Fifty universities are currently located in Ethiopia, ten of which are found in the Amhara Regional State. There are ten public universities within the region, namely, Bahir Dar, Gonder, Debre Markos, Wollo, DebireBirhan, Woldia, Debre Tabor, Injibara, Debark, and Mekidela Amba University, which are under three major clusters or classifications (Zewude et al., 2024b). Of these, Gonder and Bahirdar Universities are research universities; Debremarkos, Wollo and Debre Birhan Universities are applied universities; whereas Mekidela Amba University, Woldia, Debre-Tabour and Injibara University are comprehensive universities. To make reliable and comprehensive research data, three universities, such as Gondar University (public university), Wollo University (research university), and Woldia University (applied university) were selected and included in the study. To control for potential biases, participants were assured that responses were anonymous and would not affect their academic standing.

### 3.2. Instruments

The development process of these instruments began with the selection of instruments through a review of appropriate scientific literature as measuring constructs relevant to this study. The selection of a scale was then carefully adapted from each scale based on their relevance to the specific objective of this study. The selection criteria are aligned with the theoretical frameworks. The reliabilities (Cronbach’s alpha, composite reliability) and validities (convergent, discriminant validity, construct validity using confirmatory factor analysis) of the scales were calculated and reported in this study. A number of scales from each instrument was chosen to measure specific constructs which are targeted in this study.

#### 3.2.1. Demographics Questionnaire and Data Quality Controlling

The demographic questionnaire obtained information regarding sex, age, university batch (year), and university type. Additionally, to increase the accuracy of the data there were four attention and honesty check items included based on scientific recommendations (Meade & Craig, 2012; Verigin et al., 2019; T. Wang et al., 2024). The first item, “Please choose ‘agree’ for this question,” was used to test whether the subject was careless or inattentive. If the subject did not choose the fixed response of “agree”, their data were excluded (Meade & Craig, 2012; T. Wang et al., 2024). The next three items which were rated on a 4-point Likert scale from 1 (strongly disagree) to 4 (strongly agree)—“I answered all the questions truthfully”, “I never lied”, and “I never hid myself”—were intended to assess the honesty and truthfulness of the subject’s responses. If a person scored low (≤2 on the 4-point Likert scale) on any of these three items, their data were also excluded, as this would suggest a tendency toward self-reported deception (Meade & Craig, 2012; Verigin et al., 2019; T. Wang et al., 2024). By including these validity checks, the researchers aimed to ensure the truthfulness and reliability of the self-reported data collected through the demographic questionnaire.

#### 3.2.2. Career Self-Efficacy

Career self-efficacy was measured using the Career Decision-Making Self-Efficacy Scale-Short Form (CDSE-SF) developed by Betz et al. (1996), which is used to assess university students’ beliefs about their capacity to make career-related decisions. The CDSE-SF is composed of a total of 25 items measured with five dimensions, and in the present study the internal consistency and construct reliability, respectively, were: (a) occupational information, (b) planning, self-appraisal, goal selection, and problem-solving. 

#### 3.2.3. The Five Facet Mindfulness Questionnaire Short Form (FFMQ-SF)

The FFMQ-SF developed by Baer et al. (2006) and later modified by Hou et al. (2014) was used in this study to assess mindfulness levels. The FFMQ-SF has five sub-scales and respondents’ responses are given on a 5-point Likert scale ranging from 1 (very rarely true) to 5 (almost always true). The original version of each sub-scale with four items: acting with awareness, describing, observing, non-judging, and non-reactivity. 

#### 3.2.4. Career Adapt-Ability Short Form Five Scale (CAAS-5-SF)

For this study, we used the validated Chinese (Nye et al., 2018; Sou et al., 2021) and Switzerland (Savickas & Maggiori, 2017) versions of the CAAS 2.0 (Savickas & Porfeli, 2012). The CAAS-5-SF instrument assesses an undergraduate student’s ability to adapt to changing work environments and circumstances (Nye et al., 2018; Sou et al., 2021). Respondents’ responses are given on a 5-point Likert scale ranging from 1 (not strong) to 5 (strongest). The CAAS-5SF has five sub-scales, each with three items: concern, control, curiosity, confidence, cooperation and total.

#### 3.2.5. Basic Psychological Needs Satisfaction Scale (BPNSS-12)

Basic psychological needs satisfaction was assessed using the BPNSS-12 developed by Chen et al. (2015), consisting of 12 items, which can be found published in the *Journal of Motivation and Emotion*. A 5-point Likert scale (1 = not true at all to 5 = completely true) is used to assess undergraduate students’ satisfaction with basic psychological needs. An example item is: “I feel I have been doing what interests me.” The three-factor BPNSS-12 sub-scales in the present study demonstrated are as follows: autonomy, competence, and relatedness.

#### 3.2.6. Career Choice Scale (CCS)

The career choice scale was developed to measure the degree of an individual’s perception of their ability related to their future career path to set and achieve career goals, solve work-related problems, acquire and utilize job-related skills, cope with career challenges and setbacks, adapt to changes in the workplace, and make informed decisions (Chan, 2018). It is a measurement tool consisting of six items and a single factor scored between 1 (no confidence at all) and 5 (complete confidence) points. The scale was adapted to Taiwan by Chan (2018). 

### 3.3. Procedures of the Studies

#### 3.3.1. Data Collection System

The research undertaken engaged participants from three prominent public universities situated in the Amhara Regional State of Ethiopia. University stratification, overseen by the Ministry of Education of Ethiopia, categorized these institutions based on varying degrees of quality, objectives of the nation’s needs, and internationalization status (Zewude & Hercz, 2024). To ensure reliable data collection, an offline survey was deemed the most suitable method due to students’ limited awareness of research significance and the potential for inadvertent errors. Participants were briefed on the study’s objectives, and their involvement was entirely voluntary, with data collection spanning three months from December to February during the 2023/2024 academic year.

#### 3.3.2. Adaptation, Translation, and Validation of the Measures

Cross-cultural validation was employed to adapt and validate instruments originally developed for other cultures (Tyupa, 2011). This process ensures the suitability of the measures in a culturally diverse context like Ethiopia (Davidov et al., 2018). Various scales, such as the Career Decision-Making Self-Efficacy Scale-Short Form (CDSE-SF; Sondhi & Joshi, 2024), the Five Facet Mindfulness Questionnaire Short Form (FFMQ-SF; Baer et al., 2006; Hou et al., 2014; Zewude et al., 2024b), the Career Adapt-Ability Short Form Five Scale (CAAS-5-SF; Nye et al., 2018; Sou et al., 2021), the Basic Psychological Needs Satisfaction Scale (BPNSS-12) by Chen et al. (2015), and the Career Choice scale (CCS) by Chan (2018), were adapted and translated for use in the Ethiopian context. The recommended guidelines for cross-cultural validation were followed, including forward and backward translation, synthesis, expert review, and testing validation (Beaton et al., 2000). The adapted instruments were then pretested and validated.

After validating the measures, the study aimed to conduct a parallel mediation analysis to explore the indirect effects of independent variables on a dependent variable through a series of mediators (Hong et al., 2019). Parallel mediation analysis (PMA) is particularly useful when there is a sequential chain of mediators, where each mediator influences the next until the final mediator affects the dependent variable (Zewude et al., 2025). This analysis helps uncover the complex relationships and underlying mechanisms between variables. Confirmatory factor analysis (CFA) and structural equation modeling (SEM) were used to assess the measurement (factorial) validity and structural validity of the proposed models. Goodness-of-fit indices, such as normed chi-square, the Tucker–Lewis index, the comparative fit index, standardized root mean residual, and root mean squared error of approximation, were considered to evaluate the model fit (Hu & Bentler, 1999). CFA and SEM enhance the robustness of the analysis. The study identified BPNS and mindfulness as influential factors mediating the relationship between career self-efficacy, career adaptability, and university students’ future career choices

### 3.4. Ethics of the Research

The data-gathering process for this scholarly inquiry meticulously followed the established guidelines set forth by the American Psychological Association (APA). Before initiation, the study secured ethical approval from the ethics committee at the primary author’s academic institution, Institute IRB, with the notable reference number 301/2023. All participants were duly informed about the study’s objectives, and the procedures were executed in strict adherence to the Helsinki Declaration, encompassing compliance with regulations such as 21 CFR 50 (Protection of Human Subjects) and 21 CFR 56 (Institutional Review Boards). Throughout the research endeavor, the researchers rigorously adhered to the protocols, guidelines, and regulations stipulated by the International Research Code of Ethics.

## 4. Results

### 4.1. Descriptive Statistics, Kurtosis and Skewness

Table 2 provides the descriptive statistics, including the mean and standard deviation, for the different variables in the study. The absolute values of kurtosis and skewness for the variables (CA, CSE, CC, mindfulness, and BPNS) are within the acceptable range for normal distribution (kurtosis ≤ 4, skewness ≤ 2) recommended by Zewude et al. (2024a). In this study, the skewness values for CA, CSE, CC, mindfulness, and BPNS for university students were found to be 0.01, −0.486, −1.09, −0.745, and −0.982, respectively. The kurtosis values for these constructs for university students were also found to be −0.580, 0.188, 1.53, 2.03, and 1.30, respectively.

### 4.2. Reliability of the Employed Instruments

In this study, we confirmed the construct validity of the instruments. The results are as follows.

**Career Self-Efficacy**: The CDSE-SF is a total of 25 items measured with five dimensions and in the present study, the internal consistency and construct reliability, respectively, were: (a) occupational information (α = 0.94; CR = 0.94; AVE = 0.76); (b) planning (α = 0.93; CR = 0.93; AVE = 0.71); self-appraisal (α = 0.94; CR = 0.94; AVE = 0.75); goal selection (α = 0.95; CR = 0.95; AVE = 0.81); problem-solving (α = 0.95; CR = 0.95; AVE = 0.80); and the total scale was α = 0.94. A construct validity of the measure using CFA found that the five-factor model looked to provide an acceptable fit to the data (χ2/df = 3.44, TLI = 0.938, CFI = 0.946, RMSEA = 0.053 (95% CI = 0.040, 0.065), SRMR = 0.33). 

**The Five Facet Mindfulness Questionnaire Short Form (FFMQ-SF)**: In the present study, the five sub-scales, such as (a) acting with awareness (α = 0.95; CR = 0.95; AVE = 0.82), describing (α = 0.94; CR = 0.94; AVE = 0.79), observing (α = 0.92; CR = 0.92; AVE = 0.74), non-judging (α = 0.92; CR = 0.92; AVE = 0.74), and non-reactivity (α = 0.87; CR = 0.89; AVE = 0.67) demonstrated high internal consistency, construct reliability, and discriminant validity, respectively. The construct validity of the FFMQ-SF using CFA demonstrated that the five-factor model looked to provide an adequate fit to the current data (χ2/df = 2.99, TLI = 0.978, CFI = 0.982, RMSEA = 0.044 (95% CI = 0.040, 0.049), SRMR = 0.29). 

**Career Adapt-Ability Short Form Five Scale (CAAS-5-SF):** The CAAS-5SF has five sub-scales, each with three items, such as concern (α = 0.84), control (α = 0.86), curiosity (α = 0.82), confidence (α = 0.81), cooperation (α = 0.75) and total (α = 0.93). The original version of CAAS-5SF demonstrated high reliability and construct validity (Soares et al., 2023; Sou et al., 2021). In the present study, the five-factor CAAS-5SF sub-scales of concern (α = 0.87; CR = 0.87; AVE = 0.68), control (α = 0.91; CR = 0.91; AVE = 0.78), curiosity (α = 0.89; CR = 0.89; AVE = 0.72), confidence (α = 0.91; CR = 0.91; AVE = 0.79), and cooperation (α = 0.83; CR = 0.83; AVE = 0.62) demonstrated high internal consistency, construct reliability, and discriminant validity, respectively. The construct validity of the CAAS-5SF using CFA demonstrated an adequate fit to the current data (χ2/df = 4.03, TLI = 0.947, CFI = 0.960, RMSEA = 0.060 (95% CI = 0.054, 0.076), SRMR = 0.056). 

**Basic Psychological Needs Satisfaction Scale (BPNSS-12)**: The three-factor BPNSS-12 sub-scales in the present study demonstrated (autonomy (α = 0.89; CR = 0.91; AVE = 0.70), competence (α = 0.93; CR = 0.93; AVE = 0.77), and relatedness (α = 0.93; CR = 0.94; AVE = 0.78)) high internal consistency, construct reliability and discriminant validity, respectively. A construct validity of the measure using CFA found that the three-factor model looked to provide an excellent fit to the data (χ2/df = 4.34, TLI = 0.930, CFI = 0.946, RMSEA = 0.081(95% CI = 0.070, to 0.092), SRMR = 0.041), which confirmed an acceptable range based on the conventional criteria’s of Hu and Bentler (1999). 

**Career Choice Scale (CCS)**: The Cronbach’s α value of the scale (CCS) was found to be 0.78. In the present study, the scale demonstrated (α = 0.94; CR = 0.94; AVE = 0.73), high internal consistency, construct reliability, and discriminant validity, respectively. The confirmatory factor analysis (CFA) was performed and construct fit values showed an excellent fit in the current study (χ2/df = 3.08, TLI = 0.986, CFI = 0.991, RMSEA = 0.040 (95% CI = 0.033, 0.059), and SRMR = 0.013). 

**The Scale Overall Evaluation**: The author(s) of the study conducted several analyses to evaluate the quality of the scales used in the research. The results showed that all estimated parameters for the scales were statistically significant, indicating that the scales effectively measured the intended constructs. Additionally, the composite reliability of the scales, which assesses the internal consistency or reliability of the items within each scale, ranged from 0.83 to 0.95. These values were higher than the recommended threshold of 0.60 proposed by Fornell and Larcker (1981). This suggests that the scales demonstrated excellent internal consistency, implying that the items within each scale were highly reliable measures of the constructs.

Furthermore, the average variance extracted (AVE) for each scale, which measures the amount of variance captured by the scale’s items relative to measurement error, ranged from 0.62 to 0.82. These values exceeded the suggested threshold of 0.50 by Fornell and Larcker (1981). This indicates that the scales exhibited good convergent validity and discriminant validity, effectively measuring the intended constructs and sharing a substantial amount of variance. Therefore, based on the analysis results, it can be concluded that the four scales used in the study demonstrated convergent validity, good discriminant validity, and satisfactory internal quality. 

### 4.3. Multicollinearity Diagnostics

As seen in Table 3, there are no issues with multicollinearity in our present study, as indicated by the tolerance values of each predictor variable (CA, CSE, mindfulness and BPNS) on the criterion variable (CC) being close to those in the model. Conversely, if the tolerance values were close to zero, it would suggest a higher risk of multicollinearity (Hair et al., 2019). To assess multicollinearity, the VIF statistic should ideally fall between 0 and 5, with lower numbers being more desirable, even approaching 0 (Zewude et al., 2024b). In our study, the VIF ranged between 0.806 and 0.941, which means below 5, indicating the absence of multicollinearity. Additionally, the tolerance limits for each independent variable were all greater than or equal to 1.00, further supporting the conclusion that our independent variables were free from multicollinearity issues, as measured by VIF and tolerance.

Furthermore, we conducted the Harman single-factor test to investigate the presence of common method bias in our study. The results revealed that all constructs exhibited a common method bias rate of only 20%, which falls below (50%) the recommended fit requirements. Therefore, we concluded that our study demonstrated the absence of multicollinearity issues through the favorable tolerance values and VIF scores. The low rate of common method bias further validated the reliability of our findings.

### 4.4. Pearson Correlation Among the Study Constructs

Table 4 shows the results revealing a positive correlation between CA and CSE (r = 0.147, *p* < 0.01), BPNS (r = 0.229, *p* < 0.01), mindfulness (r = 0.110, *p* < 0.01), and CC (r = 0.252, *p* < 0.01) in university students. In addition, we found also a positive correlation between CSE and BPNS (r = 0.364, *p* < 0.01), mindfulness (r = 0.192, *p* < 0.01), and CC in the sample of students (r = 0.270, *p* < 0.01). Similarly, a positive significant correlation was found between mindfulness and BPNS and CC (r = 0.211, *p* < 0.01). Regarding the demographic factors, the gender of the participants had a significant positive correlation with CA, while it exhibited a negative correlation with CSE, mindfulness, and CC. University type also demonstrated a significant negative correlation with CA, but it displayed significant positive correlations with CSE, BPNS, mindfulness, and CC. Lastly, the batch/year of study only showed a significant positive correlation with the mindfulness of university students. Our analysis of the Pearson correlation coefficients revealed significant associations between the main constructs and variables such as gender, batch, and university type. As a result, it is necessary to further investigate the impact of socio-demographic factors on the main constructs of CA, CSE, BPNS, mindfulness, and CC. 

### 4.5. Measurement and Structural Models

Measurement and structural models were then assessed. The measurement model consisted of 5 latent constructs and 19 indicators. The CA scale had five indicators (concern, control, curiosity, confidence, and cooperation); the Career Decision-Making Self-Efficacy Scale-Short Form (CDSE-SF) also consisted of five latent indicators (occupational information, planning, self-appraisal, goal selection, and problem-solving); the Facet Mindfulness Questionnaire Short Form (FMQ-SF) consisted of five latent indicators (acting with awareness, describing, observing, non-judging and non-reactivity) and was measured by five indicators; BPNS (BPNS) was measured by three indicators (autonomy, competence, and relatedness) with a total of twelve items; and the CC Scale (CC) was a one-dimensional indicator. The measurement model demonstrated a good fit based on confirmatory factor analysis (CFA). After examining the measurement model for the CASF-5S, CDSE-SF, FFMQ-SF, BPNS-12, and CC, our next step involved assessing the overall constructs of the measurement model across various scales. This analysis encompassed model one (1) to model three (3) for university students. The result demonstrated a good fit to the data for all models (model 1 to model 3). These results indicate that the latent variables are accurately represented by their corresponding indicators. The structural model was then evaluated after a confirmed measurement model. To test the structural model, we examined the fitness of indices of the latent constructs and fitted them to the data, yielding the acceptable fitness of indices (Table 4). 

Finally, the full and the partial mediation models of the structural models were carefully analyzed, namely, CA → BPNS and mindfulness → CC (Model 1-CA); CSE → BPNS and mindfulness → CC (Model 2-CSE); and CA and CSE → BPNS and mindfulness → CC (Model 3-CA and CSE). The findings of this study revealed that the measurement models and structural models (Models 1 to 3) demonstrated an acceptable fit to the data and ensured structural fitness and measurement appropriateness. All factor loadings were significant and ranged between 0.69 and 0.95 (*p* = 0.001), indicating that the indicators effectively captured the underlying latent variables. The measurement model and structural model for university students exhibited an acceptable fit to the data (CFI > 0.60, SRMR < 0.08. RMSEA < 0.08, recommended by Oo et al. (2023)), indicating that the indicators accurately represented the latent constructs and that the relationships between the constructs were well-supported by the data. The BPNS model gave an RMSEA = 0.081, marginally exceeding the recommended threshold. The BPNS scale measures three dimensions (autonomy, competence, relatedness) with twelve items, causing complexity relative to the sample size (N = 1026). Despite the RMSEA value, other indices (CFI = 0.946 and TLI = 0.930, and SRMR = 0.041) strongly supported the model’s acceptability and interpretation (Hu & Bentler, 1999). The results of the study provide evidence for the reliability, convergent validity, discriminant validity, and construct validity of the CA, CSE mindfulness, BPNS, and CC constructs among undergraduate university students (Table 5).

### 4.6. Partial (Single) Mediation Analysis

**Figure 2:**
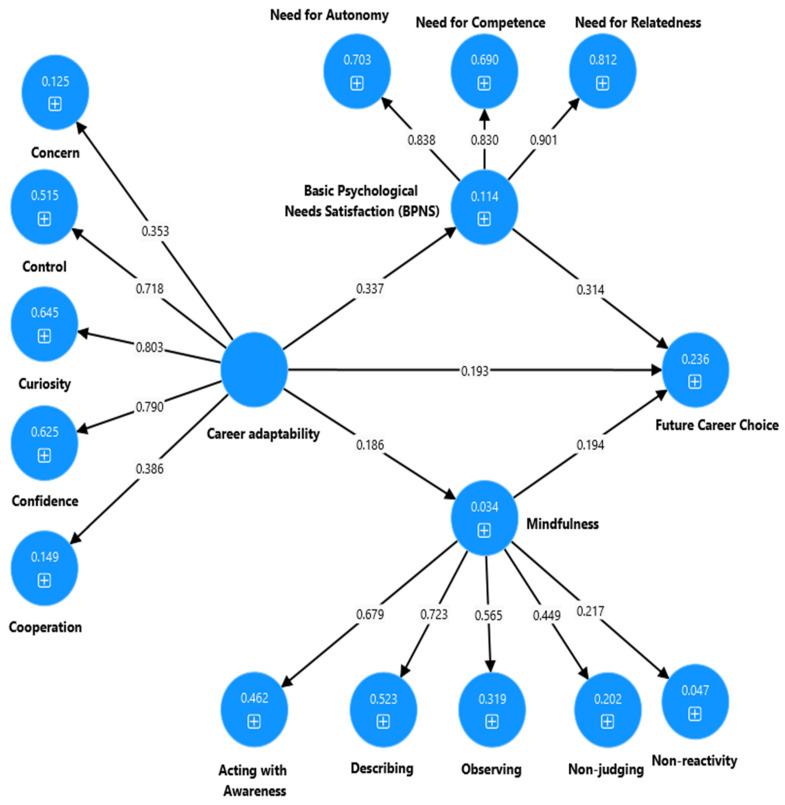
Output of the partial mediation model to explain the association between CA, BPNS, mindfulness, and CC for university students.

In the investigation of the mediating models involving BPNS and mindfulness in the interplay between CA, CSE, and CC, a targeted analysis utilizing a partial (single) mediation analysis was performed using Smart PLS software version 4.1.0.8 (Ringle et al., 2024). As shown in Figure 2, both BPNS and mindfulness together partially mediated the relationship between CA and CC for university students (β = 0.142, [bootstrapped 95% CI: 0.097, 0.189], *p* = 0.001) and it was significant in both the structural and measurement models. Specifically, the mediating effect of BPNS between CA and CC was significant (β = 0.111, [bootstrapped 95% CI: 0.076, 0.154], *p* = 0.001) for university students. In addition, mindfulness played a significant mediating effect on the relationship between CA and CC for university students (β = 0.120, [bootstrapped 95% CI: 0.065, 0.180], *p* = 0.001).

**Figure 3:**
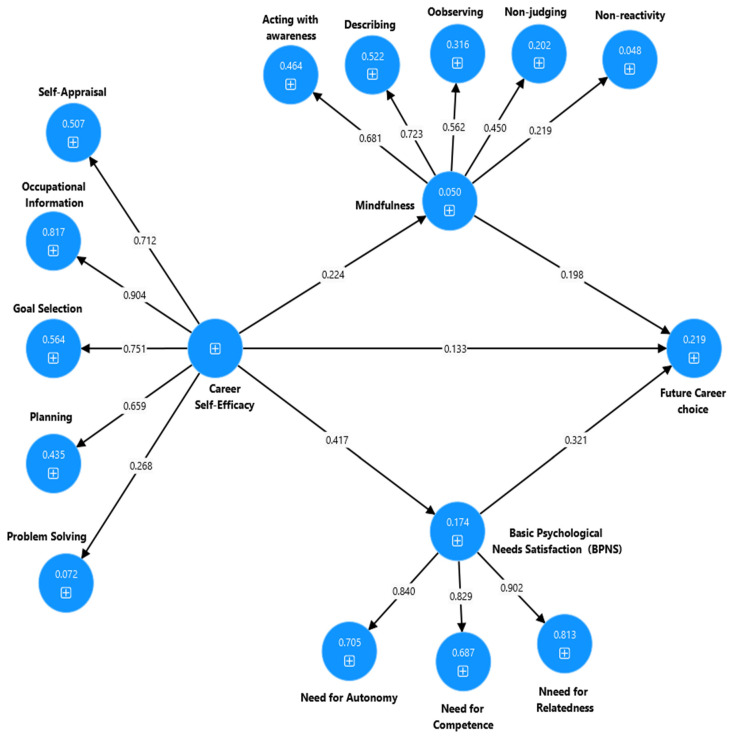
Output of the partial mediation model to explain the association between CSE, BPNS, mindfulness, and CC for university students.

Furthermore, Figure 3 shows that both BPNS and mindfulness together partially mediate the relationship between CSE and CC for university students (β = 0.178, [bootstrapped 95% CI: 0.111, 0.219], *p* = 0.002). In addition, BPNS plays a significant positive mediating role between CSE and CC (β = 0.139, [bootstrapped 95% CI: 0.103, 0.175], *p* = 0.002) for university students. Similarly, mindfulness also plays a significant mediating effect on the relationship between CSE and CC for university students (β = 0.049, [bootstrapped 95% CI: 0.028, 0.078], *p* = 0.001). 

### 4.7. Parallel Mediation Models

**Figure 4:**
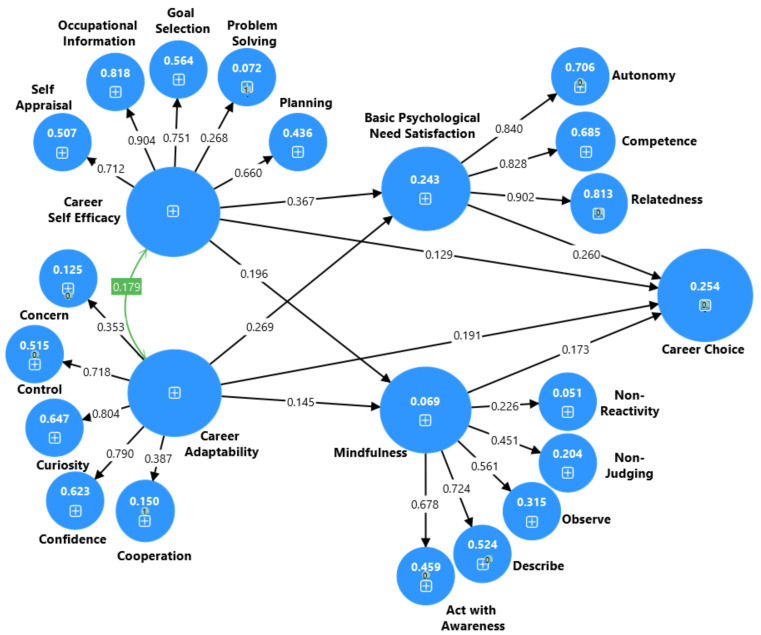
Output of a complete parallel mediation model to explain the association between CA, CSE, BPNS, mindfulness, and CC for university students.

Figure 4 shows the directionality, magnitude, and significance of the independent variables (CA, CSE, BPNS, mindfulness) influence on the dependent variable (CC) (Ringle et al., 2024). Results indicated that CA and CSE had a positive significant indirect effect on university students’ future CC through psychological needs satisfaction and mindfulness (β = 0.095 [bootstrapped 95% CI: 0.060 to 0.136], *p* = 0.001) and (β = 0.129 [bootstrapped 95% CI: 0.100 to 0.164], *p* = 0.001) for CA and CSE, respectively. University students also perceived both CA and CSE to exert a positive direct effect on BPNS (β = 0.269, [bootstrapped 95% CI: 0.197, 0.345], *p* = 0.001; β = 0.367, [bootstrapped 95% CI: 0.300, 0.430], *p* = 0.002), mindfulness (β = 0.145, [bootstrapped 95% CI: 0.063, 0.235], *p* = 0.006; β = 0.196, [bootstrapped 95% CI: 0.135, 0.263], *p* = 0.002), and CC (β = 0.191, [bootstrapped 95% CI: 0.131, 0.256], *p* = 0.002; β = 0.129, [bootstrapped 95% CI: 0.067, 0.191], *p* = 0.002), respectively. Furthermore, university students believe that BPNS (β = 0.260, [bootstrapped 95% CI: 0.188 to 0.336], *p* = 0.001) and mindfulness (β = 0.173, [bootstrapped 95% CI: 0.110 to 0.245], *p* = 0.001) positively predict students future CCs.

Then, the authors also observed a tested model aimed at investigating whether CA independently and partially influences university students’ future CC. This investigation considered the mediating factors of BPNS and mindfulness, both individually and in combination. The results revealed several significant relationships. Firstly, we found that CA had a direct positive effect on BPNS, mindfulness, and CC in the partial model with the mediating factors of BPNS and mindfulness. CA indirectly affects university students’ CC separately through BPNS and mindfulness. This suggests that CA without CSE (CSE) in a separate model predicts that students with high CA skills tend to exhibit better BPNS, mindfulness, and efficient CC. Secondly, CSE was found to have a positive direct effect on university students’ future CC through BPNS and mindfulness. This implies that students with high CSE are more likely to demonstrate better BPNS, a high level of mindfulness, and better CC. Thirdly, CSE showed a positive direct effect on students’ future CC through BPNS as well as through mindfulness, suggesting that students who have better BPNS and mindfulness skills tend to have better CC. In our parallel mediation model, the potential predictive power of CA and CSE using R^2^ values were (0.243, 24.3%) for basic psychological needs, (0.069%, 6.9%) for mindfulness, and (0.254, 25.4%) for students’ future CC. Standardized direct and indirect effects are shown in Table 6 and Table 7 for clarity.

## 5. Discussion

This research confirmed the positive relationships between career adaptability (CA) and career self-efficacy (CSE) with basic psychological needs, mindfulness, and CC for university students. This study also built a parallel mediation model to test the complex direct and indirect effects of the predictor and criterion variables. It revealed that when taking basic psychological needs and mindfulness as mediating variables, both play multiple roles in the relationship with CA, CSE, and CC. *First*, CA has a direct positive effect on basic psychological needs, mindfulness, and CC for university students. *Second*, CSE has a direct positive effect on basic psychological needs, mindfulness, and CC for university students. *Third*, basic psychological needs and mindfulness also have a positive direct effect on university students future CC. *Fourth*, through basic psychological needs and mindfulness, CA and CSE also indirectly and positively influence the CC of university students. A high level of basic psychological needs and mindfulness strategies boosts the high level of CA, CSE, and students’ future CC for university students. *Fifth*, basic psychological needs partially mediate the relationships between CA and CC as well as CSE and CC for university students. *Sixth*, mindfulness also partially plays a mediating role in CA and CC, as well as CSE and CC for university students. 

The measurement and structural models confirmed the model fitness and applicability of the proposed chain-mediation models. Basic psychological needs satisfaction and mindfulness not only influence directly but also play a mediating role among CA, CSE, and students’ future CC in university students. The findings suggest that basic psychological needs satisfaction and mindfulness play an important role in buffering and mediating the impacts of CA, CSE, and students’ future CC. From the perspectives of Bandura’s social cognitive theory, positive psychology, and career development theory, these results offer valuable insights for designing CA and CSE interventions targeting university students to inform their future career decisions and enhance their future work lives.

Specifically, consistent with several previous studies regarding the relationship among the constructs, our study revealed a significant positive correlation between CA with BPNS (Şahin & Gülşen, 2022), mindfulness (Cede & Gözen, 2021), and students future CC (Stead et al., 2022). Similarly, CSE and BPNS (Xu et al., 2023), mindfulness (Baer et al., 2006; Cede & Gözen, 2021; Yagil et al., 2023), and students’ future CC (Chan, 2018). This highlights the detrimental positive effect of CA and CSE on these important psychological factors (e.g., Baer et al., 2006; Burgueño et al., 2023; Chan, 2018; Deci & Ryan, 2000). In addition, mindfulness and future CC have a significantly positive relationship. In addition, CA and CSE are pivotal factors in shaping individuals’ career trajectories, influencing decision-making, transitions, and overall success in their professional lives. This key aspect is also in line with Lee and Jung’s (2022) study that university students with higher levels of CA tend to exhibit greater confidence in their decision-making abilities, which provides individuals with the necessary tools to navigate the ever-changing career landscape, facilitating successful transitions and enhancing overall career satisfaction leading to more informed choices and increasing the chances of success. CSE, coined from Bandura’s social cognitive theory, is crucial for individuals’ belief in their capacity to excel in future career-related tasks and it acts as a cornerstone for career decision-making, with individuals possessing higher levels of self-efficacy demonstrating more confidence and competence in their CC (Komarraju et al., 2014). Conversely, CSE empowers individuals to make informed decisions and choose paths aligned with their capabilities and aspirations, ultimately impacting their career trajectories positively (Savickas & Maggiori, 2017). Together, these constructs play essential roles in individuals’ career development, shaping their beliefs, behaviors, and ultimate success in the dynamic professional world.

The study’s key contribution is the examination of the mediating roles of BPNS and mindfulness separately and together in the relationship between CA and students’ future CC as well as in the relationship between CSE and students’ future CC. The results revealed that both BPNS and mindfulness partly and fully mediated the positive relationship between CA and students’ future CC and CSE. Our findings align with research on the detrimental effects of CA and CSE on students’ future CC and the mediating role of BPNS and mindfulness (Benlahcene et al., 2020; Hirschi & Valero, 2015; Lomas et al., 2017). Specifically, the study confirmed that higher levels of BPNS and mindfulness leads to higher levels of CA and CSE and better students’ future CCs among university students, consistent with the potential inferential and mediating role of BPNS and mindfulness in mediating these relationships and students’ future CCs (Lomas et al., 2017). Additionally, BPNS and mindfulness were found to partially mediate the relationship between CA and students’ future CC as well as CSE and students’ future CC, highlighting the importance of fostering mindfulness intervention and BPNS to mitigate the positive role of CA and CSE to foster students future CCs and to maintain healthy career development.

This study also investigated the direct effects of CA, CSE, BPNS, and mindfulness on students’ future CC. Several studies consistent with our study found that both CA and CSE are instrumental in shaping students’ future CC and have positive impacts on students’ CC, exhibited in students’ greater confidence in decision-making which leads to more informed choices, increased success chances, and boosted self-efficacy beliefs. Together, this contributes to more decisive and confident career decisions (Hirschi & Valero, 2015; Lomas et al., 2017).

Interestingly in this study, CA and CSE were found to be positive predictors of BPNS, mindfulness, and future CC. The results confirmed that BPNS and mindfulness are significant positive predictors of CC, supporting the idea that BPNS and mindfulness are positively related to CC. This finding aligns with several previous studies (e.g., Burgueño et al., 2023; Deci & Ryan, 2000; Chan, 2018; Y. Wang et al., 2019; Xu et al., 2023). The complex relationships and influences of each predictor on students’ future CC, specifically for university students, require further investigation and comprehensive analysis using a chain-mediation approach. This work has not been performed anywhere to our current knowledge. For university students, this study highlights that basic psychological needs satisfaction and mindfulness are critical positive factors that boost CA, CSE, and CC. This finding is also in line with Chan’s (2018) study that showed the importance of mindfulness for students’ career adaptability. The relationship between BPNS, mindfulness, CA, CSE, and CC has been identified, and BPNS and mindfulness can help improve CA skills, CSE, and informed CC. These findings emphasize the need for interventions that address both CA and CSE as potential positive psychological factors, and that both BPNS and mindfulness played a mediating role in enhancing students’ future CC. 

The study’s contributions include conceptual, methodological, and contextual gaps by testing a serial/chain-mediation analysis using structural equation modeling (SEM) related to the scarcity of this associational research in Ethiopian and global contexts, as well as providing new insights into the underlying mechanisms and mediators of the relationships between CA, CSE, BPNS, and mindfulness and students future CC. Universities should organize mindfulness programs (e.g., workshops, guided meditation sessions) for career counseling to help students reduce stress, enhance self-awareness, and improve decision-making clarity. Moreover, universities should set policies that reduce external pressures (family/socio-economic constraints) by expanding access to unbiased career information, internships, and job market insights. The cultural and institutional context of Ethiopia adds a unique perspective to the existing literature as it provides a perspective from a developing country. To enhance basic psychological needs satisfaction (BPNS) through autonomy-supportive teaching practices, universities should consider implementing specific mindfulness training modules and workshops. These programs can equip educators with the skills necessary to foster an environment that supports student autonomy, competence, and relatedness, which are essential components of BPNS. Research indicates that mindfulness practices can significantly improve teachers’ well-being and their ability to create supportive learning environments, ultimately benefiting student outcomes (C. Li et al., 2019).

## 6. Conclusions

This study highlights the intricate relationships among CA, CSE, BPNS, mindfulness, and future CC among university students. The findings demonstrated the significant impact of CA and CSE on enhancing BPNS and mindfulness, which in turn positively influence students’ career decisions. By establishing a parallel mediation model, the study reveals that BPNS and mindfulness not only have direct effects on CA, CSE, and CC but also act as crucial mediators in these relationships. This emphasizes the need to create environments that promote basic psychological needs satisfaction and mindfulness to bolster students’ career development.

To sum up, this body of research underscores the critical roles of CA, CSE, and basic psychological needs in shaping students’ career decision-making processes and satisfaction with their chosen paths. By cultivating adaptability, enhancing self-efficacy, and addressing fundamental psychological needs, students can navigate career transitions successfully, make informed decisions, and set the stage for a fulfilling professional journey. Educational institutions should prioritize initiatives that foster CA, self-efficacy, and mindfulness among students. Future studies should explore these relationships to enhance career guidance and support in various educational settings, equipping students to navigate the complexities of the contemporary career landscape and achieve their professional aspirations effectively.

## 7. Limitation and Future Research Direction

The study is limited to Ethiopia, and thus, the result may not be generalizable to other countries’ universities. Mindfulness training can help educators develop reflective practices that promote student engagement and emotional regulation. For instance, workshops could focus on techniques that encourage teachers to adopt autonomy-supportive behaviors, such as providing choices to students and offering rationales for tasks, which have been shown to enhance student motivation and satisfaction (Carroll et al., 2022). Additionally, integrating mindfulness practices into the curriculum can help students cultivate self-awareness and resilience, further supporting their psychological needs (Xu et al., 2025).

The findings of this study can inform educational policies and programs aimed at supporting students’ career development. It also gives a framework for creating focused interventions that advance students’ CC by improving their self-efficacy and vocational adaptability. However, self-reported data may introduce response biases, which may affect the participants’ responses. To limit this, we applied the common method bias (CMB) using Harman’s single-factor solution, VIF/tolerance, and reported reliable findings, but the use of a self-report measure and a single data source increases the potential for single method bias. While Harman’s test and others have been employed to address common method bias, it is essential to acknowledge the limitations of self-report measures in assessing psychological constructs. To further mitigate these limitations, we recommend adopting a multi-informant data approach. This involves collecting information from various sources, such as peer assessments, instructor evaluations, and objective performance metrics. By triangulating data from multiple informants, universities can obtain a more comprehensive and accurate understanding of students’ psychological needs and experiences. This approach not only reduces the potential bias inherent in self-reports but also enriches the data, providing deeper insights into the effectiveness of mindfulness interventions and teaching practices.

In addition, research indicates that different parenting styles (e.g., authoritative, authoritarian, or autonomy-supportive approaches) significantly affect students’ psychological well-being and academic success. For instance, authoritative parenting, characterized by high support and high expectations, is associated with better academic performance and psychological health compared to authoritarian or permissive styles and these parenting styles directly and indirectly influence their career choices (Zahed Zahedani et al., 2016). However, due to the scope of our research design and focus on intra-individual psychological constructs (CA, CSE, BPNS, mindfulness, and CC), we could not explicitly measure these parenting influences. Future studies could employ these parenting styles as mediators or moderators in the relations of these psychological constructs. Moreover, this study design prioritized examining the overall mediating role of mindfulness rather than its different subcomponents. We recommend for future research to investigate whether these subcomponents (e.g., non-judgment, non-activity, etc.) uniquely contribute to career adaptability or interact with other variables. To address these longitudinal meta-analytic and experimental studies, many universities are needed. Despite these limitations, we believe that this study offers a trustworthy and crucial view of CC and the factors affecting it. 

## Figures and Tables

**Table 1:** Distribution of participants in the study.

Participants	Number	Percentages
***University***		
Research university	352	34.3
Applied university	319	31.1
Comprehensive research university	355	34.6
***Batch (Year of the Study)***		
Freshman	275	26.8
Sophomore	299	29.1
Senior	452	44.1
***Gender***		
Female	495	48.2
Male	531	51.8
Total	1026	100%

**Table 2:** Descriptive statistics, kurtosis, and skewness of university students.

Variables	Minimum	Maximum	Mean	Standard Deviation	Skewness	Kurtosis
CA	22	90	60.12	11.30	0.001	0.580
CSE	25	150	93.53	22.60	−0.486	0.188
Mindfulness	20	123	81.07	15.49	−0.745	2.036
BPNS	12	74	48.61	12.71	−0.982	1.306
CC	6	42	25.79	6.31	−1.099	1.528

**Table 3:** VIF and tolerance of multicollinearity statistics on students’ CC.

Model	Standardized Coefficients	t	Sig.	Collinearity Statistics	
	Beta			VIF	Tolerance
CA	0.161	5.582	0.000	0.941	1.063
CSE	0.130	4.295	0.000	0.854	1.172
BPNS	0.265	8.473	0.000	0.806	1.241
Mindfulness	0.100	3.427	0.001	0.922	1.085

**Table 4:** Pearson correlations (r) among the socio-demographic factors and the study constructs (N = 1026).

	Variables	1	2	3	4	5	6	7	8
1	Sex	1							
2	University Type	–0.055	1						
3	Batch/Year of Study	0.046	–0.063 *	1					
4	CA	0.107 **	–0.071 *	–0.004	1				
5	CSE	–0.097 **	0.160 **	–0.051	0.147 **	1			
6	BPNS	0.014	0.090 **	0.040	0.229 **	0.364 **	1		
7	Mindfulness	–0.118 **	0.100 **	–0.077 *	0.110 **	0.192 **	0.255 **	1	
8	CC	–0.079 *	0.106 **	–0.054	0.252 **	0.270 **	0.375 **	0.211 **	1

Note: * Correlation is significant at the 0.05 level (2-tailed). ** Correlation is significant at the 0.01 level (2-tailed).

**Table 5:** Confirmatory factor analysis of the constructs using the measurement model and the structural model.

Models		Confirmatory Factorial Analysis of the Variables					
		χ^2^(df)	χ^2^/df	TLI	CFI	SRMR	RMSEA
Measurement model	CA	482 (80) *	4.03	0.947	0.963	0.056	0.060
	CSE	1705(965) *	3.43	0.938	0.946	0.033	0.053
	BPNS	680 (51) *	4.34	0.930	0.946	0.041	0.081
	Mindfulness	478 (265) *	2.99	0.978	0.982	0.029	0.044
	CC	54 (9) *	3.08	0.986	0.991	0.013	0.040
Model 1-CA	Measurement Model	3607(1234) *	2.92	0.943	0.949	0.043	0.043
	Structural Model	4376 (1307) *	3.35	0.930	0.934	0.051	0.048
Model 2-CSE	Measurement Model	5800(1799) *	3.22	0.931	0.937	0.037	0.047
	Structural Model	6617(1872) *	3.53	0.922	0.925	0.052	0.050
Model 3-CA and CSE	Measurement Model	7537 (2754) *	2.74	0.920	0.935	0.061	0.041
	Structural Model	8746(2898) *	3.02	0.918	0.921	0.068	0.044
	Rule of Thumb		≤5	≥0.90		≥0.08	≥0.08

**Note:** * *p* < 0.001, χ^2^ = chi-squared, df = degrees of freedom, TLI = Tucker–Lewis index, CFI = comparative fit index, RMSEA = root mean error square of approximation. Model 1-CA: CA → BPNS and mindfulness → CC (see Figure 2). Model 2-CSE: CSE → BPNS and mindfulness → CC (see Figure 3). Model 3-CA and CSE: CA and CSE → BPNS and mindfulness → CC (see Figure 4).

**Table 6:** Direct effects of independent variables on students’ CC using a 95% biased corrected confidence interval (N = 1026).

Standardized Direct Effect					
Predictors	Outcome Variables	Bootstrap 95% CI			
		Beta	LBC	UBC	*p*-Value
***Full Parallel Mediation: Direct Effect of CA and CSE on students’ future CC through BPNS and Mindfulness***					
CA	BPNS	0.269	0.197	0.345	0.001
CA	Mindfulness	0.145	0.063	0.235	0.006
CA	CC	0.191	0.131	0.256	0.002
CSE	BPNS	0.367	0.300	0.430	0.002
CSE	Mindfulness	0.196	0.135	0.263	0.002
CSE	CC	0.129	0.067	0.191	0.002
BPNS	CC	0.260	0.188	0.336	0.001
Mindfulness	CC	0.173	0.110	0.245	0.001
***Partial Mediation: Direct Effect of CA on students’ future CC through BPNS and Mindfulness***					
CA	BPNS	0.337	0.261	0.416	0.001
CA	Mindfulness	0.186	0.101	0.273	0.002
CA	CC	0.193	0.133	0.259	0.002
BPNS	CC	0.314	0.247	0.383	0.001
Mindfulness	CC	0.194	0.129	0.264	0.002
***Partial Mediation: Direct Effect of CSE on CC through BPNS and mindfulness***					
CSE	BPNS	0.417	0.358	0.474	0.002
CSE	Mindfulness	0.224	0.162	0.283	0.002
CSE	CC	0.133	0.070	0.196	0.002
BPNS	CC	0.321	0.243	0.393	0.002
Mindfulness	CC	0.198	0.127	0.272	0.002

**Note:** The model description of predictors, mediators and outcome variables. CI = confidence interval; LBC = lower bound, UBC = upper bound.

**Table 7:** Bootstrapping standardized indirect effect using 95%-biased corrected confidence interval predicting for university students (N = 1026).

Standardized Indirect Effect					
Models		Bootstrap 95% CI for University Students			
		Beta	LBC	UBC	*p*-Value
**Model 1-CA**		0.142	0.097	0.189	0.001
**Model 2-CSE**		0.178	0.1111	0.219	0.002
**Model 3-CA and CSE**	CA	0.095	0.060	0.136	0.001
	CSE	0.129	0.100	0.164	0.001

**Note:** CI = confidence interval, LBC = lower bound, UBC = upper bound. Model 1-CA: CA → BPNS and mindfulness → CC (see Figure 2). Model 2-CSE: CSE → BPNS and mindfulness → CC (see Figure 3). Model 3-CA and CSE: CA and CSE → BPNS and mindfulness → CC (see Figure 4).

## Data Availability

The corresponding authors hold the data sets generated and analyzed during this study and are willing to share them upon request.
